# Anti-Biofilm, Antibacterial, and Anti-Quorum Sensing Activities of Selected South African Plants Traditionally Used to Treat Diarrhoea

**DOI:** 10.1155/2022/1307801

**Published:** 2022-09-28

**Authors:** Rasheed Omotayo Adeyemo, Ibukun Michael Famuyide, Jean Paul Dzoyem, McGaw Lyndy Joy

**Affiliations:** ^1^Phytomedicine Programme, Department of Paraclinical Sciences, Faculty of Veterinary Science, University of Pretoria, Private Bag X04, Onderstepoort, Pretoria 0110, South Africa; ^2^Department of Microbiology/Immunology, Faculty of Basic Medical Science, Kampala International University, Kampala, Uganda; ^3^Department of Biochemistry, Faculty of Science, University of Dschang, Dschang, Cameroon

## Abstract

The development of resistance of microorganisms to conventional antibiotics is a major global health concern; hence, there is an increasing interest in medicinal plants as a therapeutic option. This study aimed to evaluate the antibacterial, anti-biofilm, and anti-quorum activities of crude extracts prepared using various solvents of nine indigenous South African plants used locally for the treatment of diarrhoea. The minimum inhibitory concentration (MIC) was determined using the broth microdilution method and the crystal violet assay was used to test the anti-biofilm activity of the extracts against a panel of bacteria. Anti-quorum sensing activity of the extracts was assessed via inhibition of violacein production in *Chromobacterium violaceum* ATCC 12472. Preliminary screening of extracts against *E. coli* ATCC 25922 revealed that the acetone extracts had significant activity, with MIC values ranging from 0.04 to 0.63 mg/mL. Further screening against a panel of bacterial pathogens showed that the acetone extract of *Bauhinia bowkeri* was the most active with MIC of 0.01 mg/mL against *Salmonella enteritidis*, followed by *Searsia lancea* with MIC of 0.03 mg/mL against *Bacillus cereus*. All the plant extracts prevented the attachment of biofilms by more than 50% against at least one of the tested bacteria. However, only the mature biofilm of *B*. *cereus* was susceptible to the extracts, with 98.22% eradication by *Searsia pendulina* extract. The minimum quorum sensing inhibitory concentration of the extracts ranged from 0.08 to 0.32 mg/mL with *S*. *lancea* having the most significant activity. The extract of *S*. *lancea* had the best violacein production inhibitory activity with IC_50_ value of 0.17 mg/mL. Overall, the results obtained indicate that acetone extracts of *S*. *leptodictya*, *S*. *lancea*, *S*. *batophylla*, *S*. *pendulina*, *B*. *galpinii*, and *B*. *bowkeri* possess antibacterial and anti-biofilm activities and can modulate quorum sensing through the inhibition of violacein production. Therefore, these results signify the potential of the selected plant extracts in treating diarrhoea through inhibition of bacterial growth, biofilm formation inhibition, and quorum sensing antagonism, supporting their medicinal use.

## 1. Introduction

Diarrhoea is the disruption of normal morphological and physiological functioning of the gastrointestinal tract resulting in an abnormal increase in stool volume, frequency, and fluidity. Diarrhoea is a major cause of infant death after pneumonia, especially in developing countries, accounting for about 10% of total deaths in children [1]. It is the third highest cause of death in South Africa. In animal production, diarrhoea causes significant loss in yield, leading to major economic setbacks [2]. Bacterial infectious diarrhoea is primarily caused by members of the Enterobacteriaceae, a family of Gram-negative opportunistic bacteria responsible for enormous infections [3]. The majority form part of the normal intestinal flora. *Escherichia coli* is the most frequently isolated microbe from enteric diseases and is referred to as diarrhoeagenic *Escherichia coli* (DEC) [4]. Over 80% of human microbial infections are associated with a syndicate of bacterial cells called biofilms. Biofilms are complex structures formed when bacterial colonies group together within an extracellular matrix, leading to the irreversible attachment to biotic and abiotic components, providing protection, and aiding in antimicrobial resistance [5]. This group of cells has clinical importance in preventing the uptake antibiotics and avoiding the effect of harsh environmental conditions. Biofilms enhance bacterial growth, antibiotic resistance, immune cell evasion, and genetic material transfer [5]. Some bacteria species like *E*. *coli* and *Shigella* species have reportedly been linked to diarrhoea virulence gene expression and biofilm formation [6]. Biofilms are closely linked to intercellular communication, otherwise called quorum sensing (QS). With the help of diffusible signaling molecule called autoinducers, gene expressions are regulated, making them difficult to eradicate by antimicrobial agents or host immune cells [7]. Hence, there is a need for alternative or complementary remedies to combat the menace of antibiotic resistance assisted by biofilm formation.

Plants have been used traditionally to treat infectious diseases for centuries. Antimicrobial resistance to conventional antibiotics, poor medical facilities, and poverty, especially in low-income countries, contributed to the use of medicinal plants as therapy [8]. Medicinal plants contain bioactive secondary metabolites like alkaloids, saponins, tannins, and flavonoids with numerous therapeutic functions, such as antibacterial, anti-inflammatory, antioxidant, and immune cells stimulation [9].

Plant extracts have been widely tested for direct antibacterial activity; however, no novel antibiotics derived from a plant have succeeded to become commercialised. There is, therefore, a necessity to look beyond microbial growth destruction by plants but rather the use of plants as biofilm disruptors and quorum sensing inhibitors, thus promoting eradication of infections without resulting development of antibiotic resistance. However, there is a need for empirical investigations to establish the effectiveness of plant extracts. *Brachylaena transvaalensis*, *Searsia batophylla*, *S*. *pendulina*, *S*. *leptodictya*, *S*. *lancea*, *S*. *gueinzii*, *Bauhinia galpinii*, *B*. *bowkeri*, and *B*. *variegata* were selected based on ethnobotanical records, previous findings of antimicrobial activity in our laboratory, and available published information on the plants. This study aimed to evaluate the antibacterial, anti-biofilm, and anti-quorum sensing activities of these indigenous South African plants.

## 2. Materials and Methods

### 2.1. Plant Material and Extraction

Leaves of the selected plants were collected at the Lowveld National Botanical Gardens in Nelspruit, Mpumalanga, South Africa, in July 2019. Some plant pictures are provided in Supplementary Material (S1). Voucher specimens were prepared and deposited in the H.G.W.J. Schweickerdt Herbarium of the University of Pretoria and voucher specimen numbers (PRU) were obtained (Table 1). Two voucher specimens were lodged in the National Herbarium, South African National Biodiversity Institute (SANBI) in Pretoria, with PRE voucher specimen numbers. The collected plant materials were dried in a well-ventilated room at room temperature and ground into powder using a Janke and Künkel Model A10 mill. The powders were stored in an air-tight polythene sack and kept in the dark until use. Acetone, ethanol, 70% methanol, methanol:dichloromethane (50 : 50), and hot water were used as solvents for extraction. Ten grams of powdered plant leaves was soaked separately in 100 mL of respective solvent. After 24 h, the supernatants were filtered through Whatman No. 1 filter paper into previously weighed glass jars. This process was repeated thrice on the same plant material. The filtrates were then dried under a stream of cold air and extract yields were calculated.

### 2.2. Antibacterial Screening

#### 2.2.1. Bacterial Strains and Culture Conditions

Strains of *Escherichia coli* O157: H7 (ATCC 43888), *E. coli* (ATCC 25922), *E. coli* (ATCC 35218), *Bacillus cereus* (ATCC 21366), *Staphylococcus aureus* (ATCC 29213), *Salmonella enterica* subsp. *enterica* serovar Typhimurium (*S. typhimurium*, ATCC 39183)*, Enterococcus faecalis* (ATCC 29212), *Pseudomonas aeruginosa* (ATCC 27853), *Salmonella enterica* subsp. enterica serovar Enteritidis (*S*. *enteritidis*, ATCC 13076) were used for the antibacterial assay. They were maintained in Mueller–Hinton (MH) agar.

#### 2.2.2. Determination of Minimum Inhibitory Concentration (MIC)

A simple twofold serial dilution microplate method was used to determine the minimum inhibitory concentration (MIC) [25]. Bacterial cultures grown overnight in MH broth (Sigma Aldrich, SA) were adjusted to McFarland standard 0.5, equivalent to 1.5 × 10^8^ CFU/mL. A 100 *μ*L aliquot of sterile distilled water was added to all the wells of a 96-well microtitre plate. The prepared extracts (10 mg/mL stock concentrations) were added to the first row of the microplate and serially diluted in a 1 : 1 ratio. After that, 100 *μ*L of adjusted bacterial cultures was added to each well. The bacteria were exposed to the extracts of final concentrations ranging between 2.5 and 0.01 mg/mL. Acetone and gentamicin served as negative and positive controls, respectively. The plates were then incubated at 37°C for 18–24 h. Following incubation, 40 *μ*L (0.2 mg/mL) of p-iodonitrotetrazolium violet (INT) was added to each well and incubated for 1 h. The MIC was taken as the lowest extract concentration to show growth inhibition, visible in terms of a decrease in red colour generated by conversion of the INT to a red product by actively respiring bacteria. *E*. *coli* ATCC 25922 was used to evaluate the preliminary antibacterial potential of all the nine plants extracted with five solvents. The most extracts were selected for further screening against the other bacterial strains.

### 2.3. Cytotoxic Activity

The cytotoxic evaluation of the acetone plant extracts was done using the MTT [3-(4, 5-dimethylthiazol-2-yl)-2,5-diphenyltetrazolium bromide] assay [26] modified by McGaw et al. [15] on Vero (African green monkey kidney) cells (ATCC® CCL-81™). Cell growth inhibition for each extract was expressed in terms of LC_50_ values, defined as the concentration that caused 50% cell lethality. The experiments were carried out in triplicate and repeated thrice. The selectivity index (SI) values of extracts were calculated by dividing the LC_50_ values by the MIC values (SI = LC_50_/MIC).

### 2.4. Biofilm Forming Ability Assay

The ability of the bacteria to form biofilm was determined using the modified method of [27]. Briefly, 0.5 McFarland standard was prepared from an overnight culture of test bacteria (approximately 1.5 × 10^8^ CFU/mL) grown in Mueller–Hinton broth supplemented with 2% glucose. The standardised bacteria were further diluted 1 : 100 in culture media to obtain an approximately 1.5 × 10^6^ CFU/mL inoculum. A 100 *μ*L aliquot of the diluted inoculum was dispensed into the well, and 100 *μ*L of the culture medium was added. The plates were covered and incubated for 24 h and 48 h. The biofilm formation of the bacteria was determined quantitatively using a crystal violet stain. The plates were washed gently three times with sterile distilled water to eliminate planktonic cells. The plates were dried at 60°C for 45 min. Sessile cells were stained with 100 *μ*L of 0.1% crystal violet for 15 min. The plates were washed to remove excess stain. A 150 *μ*L aliquot of ethanol was added to destain the crystal violet bound cells attached to the wells; then 100 *μ*L of the destained ethanol was transferred into a fresh microplate and absorbance was read at 590 nm wavelength. The biofilm-forming ability was then classified based on the following: (a) non-biofilm former if OD_test_ ≤ ODc, (b) weak biofilm former if ODc < OD_test_ ≤ 2 × ODc, (c) moderate biofilm former if 2 × ODc < OD_test_ ≤ 4 × ODc, and (d) strong biofilm former if OD_test_ > 4 × ODc, where ODc is the mean OD_media ctrl_ + (3 × standard deviation of OD_media ctrl_) and OD_test_ is the mean optical density of the tested bacterial strain OD_test_ − OD_media ctrl._ Only the bacteria with moderate-to-strong biofilm forming capacity were considered for biofilm formation inhibition and eradication of preformed biofilm tests.

### 2.5. Anti-Biofilm Assay

#### 2.5.1. Inhibition of Biofilm Formation

The method of Stefanović [27] was used to investigate the ability of the acetone extracts to prevent the formation of bacterial cell mass and attachment. Briefly, 100 *μ*L (at half MIC concentration) of plant extracts and antibiotic was added in twelve replicates into the wells of 96-well microtitre plates. Then, 100 *μ*L aliquots of standardised concentration of bacterial cultures (OD_590nm_ = 0.02 equivalent to 1.0 × 10^6^ CFU/mL) grown in Mueller–Hinton broth supplemented with 2% glucose was added and incubated at 37°C for 24 h. Following incubation, the biofilm biomass was assayed using the crystal violet (CV) staining assay as described above for the biofilm formation assay. The percentage of biofilm inhibition was determined using the following formula:

(1) $$\begin{array}{c} \%\text{ Inhibition}=\left[\frac{\left({\text{OD}}_{\text{Negavtive control}}-{\text{OD}}_{\text{Sample}}\right)}{{\text{OD}}_{\text{Negavtive control}}}\right]\times 100. \end{array}$$

Biofilm inhibition was rated between 0 and 100%. Values below 0% were categorised as biofilm growth enhancement; between 0–50% indicated weak anti-biofilm activity, and above 50% represented good biofilm inhibition.

#### 2.5.2. Eradication of Preformed Biofilm

The ability of plant extracts to prevent the further formation and/or destruction of cell mass was also investigated. A standardised concentration of bacterial cell cultures (100 *μ*L) with OD_590_ = 0.02 (1.0 × 10^6^ CFU/mL) of test bacteria were aliquoted into flat bottomed 96-well microtitre plates and incubated at 37°C for 24 h without shaking. This was followed by adding 100 *μ*L aliquots of plant extracts and antibiotic (half-MIC) into the wells of a 96-well microtitre plate. The plates were further incubated at 37°C for 24 h. The biofilm biomass was quantified, and the percentage of biofilm eradication was determined as described above.

### 2.6. Anti-Quorum Sensing

#### 2.6.1. Inoculum Preparation

A single colony of the pigment-producing bacterial strain *Chromobacterium violaceum* ATCC 12472 was cultured in Luria-Bertani (LB) broth was inoculated into 10 mL of LB broth, cultured overnight in a shaker incubator at 30°C with shaking at 0.76 g. The working bacterial suspension was prepared by further diluting the overnight grown culture with LB broth to obtain an absorbance of 0.1 ± 0.02 at a wavelength of 590 nm to match McFarland standard 0.5 (1.5 × 10^8^).

#### 2.6.2. Quantitative Detection of Violacein Inhibition in the Presence of Plant Extracts

Using the method of [28], varying concentrations of plant extracts ranging from 2.5 to 0.02 mg/mL were added to eight of ten test tubes containing 5 mL of LB broth. Then, 100 *μ*L of inoculum was added to each test tube. Acetone and vanillin were added to different test tubes as negative and positive controls, respectively. The last test tube was not treated; this served as the culture control and stands as the reference to determining the percentage of violacein inhibition. All the tubes were incubated at 30°C overnight, shaking at 0.76 g. Anti-quorum sensing was evaluated based on the growth of the biosensor organism and the reduction of purple pigment production in the test tubes containing culture and extract of different concentrations. The lowest extract concentration with visible growth (turbid) and no purple pigment production was interpreted as the minimum quorum sensing inhibitory concentration (MQSIC). This was further confirmed by aliquoting medium from a test tube without turbidity and purple colouration onto an LB agar plate and incubating for 24 h to detect visible growth.

#### 2.6.3. Violacein Detection

After incubation as described above, a 1 mL aliquot was transferred from each test tube to a 15 mL centrifuge tube and centrifuged at 978.26 × *g* for 10 min to allow the violacein bacteria to form a pellet, and the supernatant was discarded. The pellets in the test tubes were resuspended in DMSO and vortexed until the pellet was completely solubilised. The tubes were centrifuged again at 978.26 × *g* for 7 min to separate the bacteria from the solution. Then, 200 *μ*L of the supernatant in each of the tubes was dispensed in wells of a 96-well microtitre plate in duplicate, and the absorbance was measured at 595 nm using a BioTek microplate reader. The percentage of violacein inhibition was calculated using the below formula.

(2) $$\begin{array}{c} \left(1\right):\%\text{ Violacein Inhibition }\%\text{ Inhibition}=\left[\frac{\left({\text{OD}}_{\text{control}}-{\text{OD}}_{\text{test}}\right)}{{\text{OD}}_{\text{control}}}\right]\times 100. \end{array}$$

The extract concentrations at which 50% of the violacein produced was inhibited (IC50) were obtained using a regression line between the % violacein inhibition and their respective concentrations.

### 2.7. Statistical Analysis

**Figure 1:**
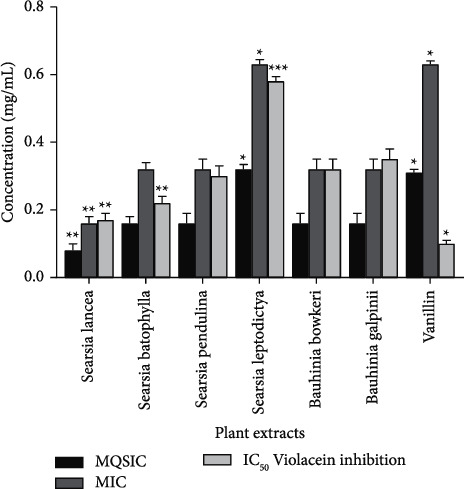
Quorum sensing inhibitory activity of acetone extracts in *Chromobacterium violaceum*. MQSIC: minimum quorum sensing inhibitory concentration, MIC: minimum inhibitory concentration, and IC_50_: 50% inhibitory concentration of violacein production.

Data were entered and collated in Microsoft Excel 356 version, and GraphPad Prism version 6.0 was used for data analysis using one-way analysis of variance (ANOVA) and Tukey's post hoc test where appropriate, with a significance level of *p* < 0.0001 and *p* < 0.05 in Table 2 and Figure 1, respectively.

## 3. Results

The characteristics of the investigated plant species are presented in Table 1.

### 3.1. Minimum Inhibitory Concentration (MIC)

The preliminary antibacterial activity of the plants extracted with five different solvents is recorded in Table 3. The MIC values against the *E*. *coli* ATCC 25922 strain ranged from 0.04 to 0.37 mg/mL for acetone extracts, 0.08 to 1.87 mg/mL for ethanol extracts, 0.32 to 2.5 mg/mL for hot water extracts, 0.29 to 2.50 mg/mL for methanol/dichloromethane extracts, and 0.16 to 2.50 mg/mL for methanol/water extracts. Therefore, the acetone extracts that were the most active against *E*. *coli* ATCC 25922 were selected for further antibacterial screening against eight other bacterial strains related to those implicated in causing diarrhoea. The data presented in Table 2 showed that *B*. *bowkeri* was the most active with MIC = 0.01 mg/mL against *Salmonella* enteritidis, followed by *S*. *lancea* which had an MIC value of 0.03 mg/mL against *Bacillus cereus,* while *S. pendulina* had MIC = 0.05 mg/mL against *E*. *coli* (ATCC 38152). In addition, *B*. *cereus* was the most resistant strain, susceptible to only *S*. *lancea* and *S*. *leptodictya* at 0.03 and 0.16 mg/mL, respectively.

### 3.2. Cytotoxicity and Selectivity Index of Tested Extracts

Compared to doxorubicin, all the plant extracts had significantly higher LC_50_ values against the Vero kidney cell line (Table 2). The LC_50_ values varied from 0.03 mg/mL to >1 mg/mL for extracts. *B*. *galpinii* was the least cytotoxic with LC_50_ > 1 mg/mL, followed by *B*. *bowkeri* with 0.51 mg/mL, while *S*. *pendulina* had the lowest value of 0.03 mg/mL, indicating the highest toxicity to the cells.

### 3.3. Biofilm Forming Ability

**Figure 2:**
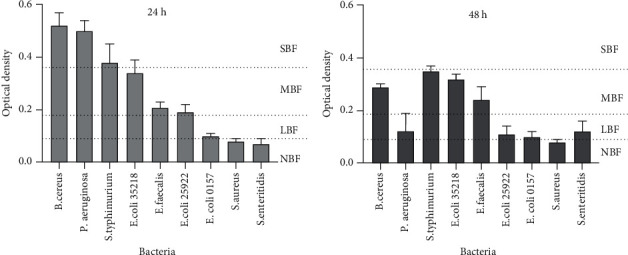
Biofilm formation ability of tested bacteria at 24 h and 48 h NBF = non-biofilm former, LBF = low biofilm former, MBF = moderate biofilm former, and SBF = strong biofilm former.

The nine bacterial strains used in this study were evaluated for their biofilm-forming capacity at two different incubation times, 24 h and 48 h. Results presented in Figure 2 show that, after 24 h, seven of the nine bacteria were able to form biofilm. Three strains (*B*. *cereus*, *P*. *aeruginosa*, and *S*. *typhimurium*) were classified as strong biofilm formers. Three strains (*E*. *coli* ATCC 35218, *E*. *faecalis*, and *E*. *coli* ATCC 25922) were moderate biofilm formers, while *E*. *coli* 0157: H7 was the only poor biofilm-forming strain. *B*. *cereus* and *P*. *aeruginosa* had the strongest biofilm-forming ability with optical densities of 0.52 and 0.50 at wavelength 590 nm after 24 h and 48 h, respectively.

After 48 h incubation, none of the bacterial strains appeared to be strong biofilm formers since all the recorded biofilm formation abilities were below the threshold of strong biofilm former capacity. *B*. *cereus*, *S. typhimurium*, *E*. *faecalis*, and *E*. *coli* 35218 were moderate biofilm former. Others were either low or no biofilm former.

### 3.4. Anti-Biofilm Activity

The anti-biofilm effect of the acetone extracts was investigated using moderate and strong biofilm forming strains. The percentage of biofilm inhibition and eradication is represented in Table 4. An inhibition percentage above 50% was considered as good anti-biofilm activity, while those with an inhibition percentage between 0 and 50% were considered poor anti-biofilm activity, and values <0 were regarded as having no anti-biofilm activity and instead considered as biofilm formation enhancers [29].

All the extracts had various levels of biofilm inhibitory activity against the bacteria. The extracts showed more than 50% biofilm inhibition against *S*. *typhimurium*, *P*. *aeruginosa*, and *E*. *coli* ATCC 25922. On the other hand, there was no biofilm formation inhibition >50% of *E*. *coli* 35218 biofilm by all the plant extracts. Except for *S*. *lancea* and *B*. *galpinii* that showed poor biofilm inhibitory activity, all other extracts had good biofilm inhibitory activity against *B*. *cereus* biofilm.

In the biofilm eradication test (treatment after 24 h), negative percentage inhibition of plant extracts was noted for five out of six tested bacteria. However, all the extracts had good biofilm eradication activity against *B*. *cereus* strains. Also, *S*. *leptodictya* had good biofilm eradication activity against *P*. *aeruginosa* with a percentage of biofilm eradication of 79.85%. However, *S*. *pendulina*, *S*. *lancea*, *B*. *galpinii*, and *B*. *bowkeri* had weak biofilm eradication activity against *S. typhimurium* with percentages lower than 50. Similar results were obtained with *S*. *batophylla* against *E*. *faecalis* and *B*. *galpinii* against *E*. *coli* ATCC 35218 (Table 4).

### 3.5. Quorum Sensing Inhibition

The ability of plant extracts to inhibit quorum sensing (QSI) was tested using a biosensor bacterium, *Chromobacterium violaceum*. The minimum quorum sensing inhibition concentration (MQSIC) was defined as the lowest concentration characterised by growth (turbidity) and no purple ring formation indicating bacterial growth without violacein pigment. All the plant extracts showed inhibition of quorum sensing at varying concentrations by inhibiting violacein production. *S. lancea* had the most significant MQSIC of 0.08 mg/mL (Figure 1). All other plants had MQSIC lower than vanillin (positive control), except *S. leptodictya*. The minimum inhibitory concentration was taken as the lowest concentration, characterised by no growth or turbidity and purple ring formation. *S. lancea* had the lowest MIC value of 0.16 mg/mL, while *S. leptodictya* had the highest MIC value of 0.63 mg/mL (Figure 1).

The plant extracts were tested against *C. violaceum* to determine the concentration having 50% violacein production inhibition (IC_50_). The IC_50_ value for all the plant extracts was obtained from the standard graph of concentration against % violacein inhibition and thus ranged between 0.17 and 0.58 mg/mL (Figure 1). The lower the IC_50_ value, the better the ability to inhibit violacein production. Statistically, *S*. *lancea* (IC_50_ = 0.17 mg/mL) and *S. batophylla* (IC_50_ = 0.22 mg/mL) had the best ability to prevent the production of violacein among the plant extracts, while *S*. *leptodictya* had the least violacein inhibition (IC_50_ = 0.58 mg/mL).

## 4. Discussion

Many conventional interventions such as oral rehydration therapy (ORT), antisecretory, or pro-absorptive agents and probiotics (post antibiotics administration) are used to treat diarrhoea. However, medicinal plants have also been used to treat various medical conditions, including diarrhoea [19]. Research interest in plant products has been increasing because of growing antibiotic resistance, which is often linked with biofilm formation [5]. Numerous biologically active phytochemicals in plants that target different microbial metabolic pathways have the potential to inhibit microbial growth and survival [30]. Despite the antimicrobial potency of medicinal plant products, there are scarce in-depth analyses of their ability to inhibit quorum sensing as well as biofilm formation due to accumulation of extracellular polymeric substances [31]. Therefore, this study aimed to evaluate the selected plants' antimicrobial, anti-biofilm, and anti-quorum sensing potential.

In this study, nine indigenous South African plants were selected based on ethnobotanical records of use in treating diarrhoea and antibacterial activity in preliminary studies.

*E. coli* ATCC 25922, a recommended reference strain for susceptibility testing, and other bacteria based on their involvement in diarrhoea episodes and ability to form biofilms were selected in this study [1, 3, 32].

The selected plants were screened for antibacterial, anti-biofilm, and quorum-sensing inhibition activities. Five different solvents with varying polarities were used for extraction and extracts were tested against the *E. coli* ATCC 25922 reference strain to determine the extractant with promising antibacterial activity. Acetone extracts had the most significant antibacterial activity against the tested bacteria and thus conform with the previous report on acetone as the most potent extractant for the screening and isolation of antimicrobial components from plants [33]. According to Eloff [34], an MIC value of ˂0.02 mg/mL is regarded as outstanding activity, 0.021–0.04 mg/mL as excellent activity, 0.041–0.08 as very good activity, 0.081–0.16 mg/mL as good activity, and 0.16 ≤ MIC ≤ 0.32 mg/mL as moderate or average activity, while MIC values above 0.32 are considered weak activity. All the acetone plant extracts had good antibacterial activity against all three of the *E. coli* strains, with only *S. batophylla* having a slightly weaker MIC value (0.18 mg/mL) above the cut-off point (0.16 mg/mL) against *E. coli* 0157: H7. The acetone extract of *B. bowkeri* had outstanding antibacterial activity against *S. enteritidis*, an organism that causes gastrointestinal disorders [35]. The antibacterial potency of *B. bowkeri* in this study was better when compared with previous findings [19]. This may be due to variation in the phytochemical constituents of the plant because of the difference in geographical location and period of the year the leaves were harvested for investigation. Little information is available on the antibacterial activity of some of the plants; however, petroleum ether, dichloromethane, ethyl acetate, and water extracts of *S. lancea* have been reported to possess good bacterial inhibitory activity when tested against *Bacillus subtilis, Escherichia coli, Klebsiella pneumoniae*, and *Staphylococcus aureus* [36]. The acetone extract of *Bauhinia galpinii* has also been reported to have good antibacterial and antifungal activities and is known to be phenolic rich in content, which probably accounts for the antimicrobial action [19]; this supports the outcome of this work.

For medicinal plants to have clinical relevance, the preparation should be selective in its toxicity. Therefore, a cell-based *in vitro* assay for cytotoxic evaluation against Vero monkey kidney cells was used to determine the toxicity of the plant extracts. Our findings showed that the toxicity value obtained for all the plant extracts was above the toxic cut-off level of 0.02 mg/mL [37]. The toxicity values were statistically lower compared to doxorubicin, a toxic drug. This suggests that the observed antibacterial activity of the plant acetone extracts may not be due to a toxic metabolic effect.

The selectivity index (SI) is the ratio of toxicity to bioactivity and is often used to evaluate the degree of selective activity of a substance. SI values greater than 1 indicate that there is greater toxicity against bacteria or infectious agents than to host cells. Preparations with SI values greater than 10 are often considered to be valuable in pursing product development [38]. The antibacterial activity of acetone extracts of both *B. galpinii* and *B. bowkeri* were excellent and coupled with low cytotoxicity, thus revealing good prospects for product development. Excellent SI values above 25 for these two plant extracts were obtained against *S.* enteritidis, which is extremely promising and deserving of further research.

Developing a nonbiocidal strategy to combating microbial infections is of paramount importance because the use of antibiotics commonly leads to drug resistance, which is of global medical concern. Biofilm-forming bacteria have shown resistance to broad-spectrum antibiotics, making the treatment of biofilm-related infections very difficult [39]. The findings of [6] showed a link between biofilm formation and virulence gene expression that could enhance attachment of diarrhoea pathogens to mucosal surfaces of the large and small intestines, resulting in immune cells evasion and prolonged infectious diarrhoea [40]. In this study, the optimum biofilm formation time for the bacterial strains studied was 24 h. This result is contrary to the finding of [41] who found that three days of incubation led to the highest formation of biofilms for *Pseudomonas aeruginosa*. This may be due to loss of exopolysaccharides, which activates biofilm detachment from surface wall or because of the different strains used [39]. *S. enteritidis*, *E. coli* O157: H7, and *S. aureus* did not form substantial biofilms. Our aim was to determine the ability of the extracts to either inhibit biofilm formation or to eradicate preformed biofilms, or both, so a concentration of half-MIC was used to ensure that the experiment was conducted at appropriate concentrations that did not completely inhibit bacterial cell growth. All plant extracts showed good biofilm inhibition activity; however, most of them were unable to destroy preformed biofilm but rather promoted further development of the established biofilms (negative % inhibition values). Only *B. cereus* preformed biofilm was eradicated with values >50% by all the plant extracts, except *B. galpinii.* Our anti-biofilm results showed that prevention of biofilm formation is easier than eliminating the existing biofilm. A similar observation was also made by Erhabor and colleagues in their study of *in vitro* bioactivity of *Combretum elaeagnoides* leaf extract against selected foodborne pathogens [42]. This could be due to the ability of plant extracts to curtail binding forces that promote cell attachments as suggested by [43]. According to a report by Taufiq and Darah [44], the negative value suggests that the bacteria reacted to the change in the environment, consequently producing a large amount of biofilm to annul the effect of the perceived unfavourable environmental condition. In addition, it is also suggested that the difficulty in total eradication of biofilm is because the consortium of microbial growth is formed by the interaction of multiple species. The biofilm inhibition results, thus, suggest that some of the selected plants could be considered in the design of a good alternative therapy for preventing microbial colonization of surfaces and epithelial layers prior to infections. It is, therefore, necessary to further investigate with clinical trials to determine their probable mechanisms of biofilm inhibition.

Quorum sensing (QS) is a bacterial intercellular communication system that allows the control of specific processes such as biofilm formation [45]. QS has been linked to some virulence expression in both Gram-positive and Gram-negative bacteria. Gram-positive bacteria often secrete autoinducer peptides (AIPs), which in high concentration activate genes' expression, such as toxin and degradative enzymes. Gram-negative bacteria, on the other hand, usually produce autoinducer homoserine lactones (AHLs), with increased concentration in the bacteria environment that promotes the expression of specific virulence genes, such as adhesins and proteases [46]. Therefore, targeting this system is another way to curtail infectious agents' propagation. Therefore, we also investigated the ability of the acetone extracts to interfere with QS signalling process. The tested acetone extracts were able to disturb the quorum sensing processes mediated by the inhibition of violacein production. All the plant extracts except *S. leptodictya* had minimum quorum sensing inhibitory concentration (MQSIC) values that were statistically better than vanillin, the positive control at a significant level of *p* < 0.05. The recorded MQSIC values were lower than their respective MICs; this implies that the QSI was not because of cell growth inhibition; it rather indicates that the plant extracts could regulate virulence factors by inhibiting violacein pigment formation at sub-MIC. Previous research findings have reported anti-quorum sensing (AQS) activity of medicinal plants against *C. violaceum* and other bacteria [47]. *B. galpinii* and *B. bowkeri* acetone extracts previously reported to contain phenolic compounds; compounds rich in phenol have been shown to be capable of inhibiting the synthesis of *N*-decanoyl-homoserine lactone, downregulate QS mediated metabolite (ethanolamine), reduce production of violacein and haemolysin, repress QS-related gene expression (*cvil* and *cviR*) in *C. violaceum* [48].

Thus, QS and biofilm formation inhibition ability of the studied plant extracts may play an important role in reducing bacterial biofilm formation and therefore mitigate diarrheal infections as well as the development of antimicrobial resistance. However, there is a need to further investigate the exact mechanism of quorum signal inhibition as well as the compounds responsible for the observed activity.

## 5. Conclusion

Acetone extracts of *Searsia leptodictya*, *S. lancea*, *S. batophylla*, *S. pendulina*, *Bauhinia galpinii*, and *B. bowkeri* had very good antibacterial activity against a panel of bacteria implicated in causing diarrhoea symptoms. In general, of all the different solvents used to extract plant material, acetone was the most successful in extracting antibacterial compounds. *B. bowkeri* and *B. galpinii* had excellent selectivity index values of 50.75 and 25.18, respectively, against *S*. *enteritidis* and are, thus, prospective candidates for product development. This study also showed that the acetone extracts of the selected plants had significant inhibitory activity against biofilm formation and quorum sensing mediated violacein pigment production at sub-MIC values. Disruption of preformed biofilms was difficult to achieve, indicating that the plant extracts had little efficacy against established bacterial biofilms. The plants with good activity have the potential to be developed as antibacterial remedies, but further studies, particularly *in vivo*, are recommended to investigate potential pharmaceutical applications.

Purification and characterisation of the bioactive compounds from the most promising plant species, including antibacterial, anti-biofilm, and anti-quorum sensing principles, would be useful. In addition to possibly serving as framework molecules for development of novel chemicals to treat diarrhoea and related symptoms, these may serve as potential chemical markers that can be used to standardise plant-based preparations derived from the plant species of interest.

## Figures and Tables

**Table 1:** Characteristics of the plant species investigated.

Plant name (family name)	Common name	Traditional use	Part used	Previous pharmacological activities	Voucher number
*Searsia pendulina* (Jacq.) Moffett (Anacardiaceae)	Witkaree (Afrikaans), garas (nama), mosilabele (South Sotho)	Stomach ailments, enema in children [10]	Leaves	Cytotoxicity, antioxidant, and antimicrobial activities [11]	PRU 127997
*Searsia leptodictya* (Diels) T. S. Yi, A. J. Mill. & J. Wen (Anacardiaceae)	Mountain karee (English), klipkaree (Afrikaans), mohlwehlwe (N. Sotho)	Gastrointestinal disorder [12]	Leaves	Cytotoxicity, antioxidant, and antimicrobial activities [11]	PRU 70151
*Searsia gueinzii* (Sond.) F. A Barkley (Anacardiaceae)	Thorny karee (English)	Gastrointestinal infections [13]	Root	Mutagenicity and antimutagenicity [13]	PRE 1004257
*Searsia lancea* (L.f.) F. A. Barkley (Anacardiaceae)	Rooikaree (Afrikaans), mokalabata (N. Sotho), inhlangutshane (Siswati), mosilabele (Thwana and S. Sotho) mushakaladza (Venda)	Diarrhoea and gall sickness [14]	Bark and leaves	Antibacterial, antihelmintic, and cytotoxicity [15]	PRU 126859
*Searsia batophylla* (Codd) Moffett (Anacardiaceae)	Bramble currant (English), Braamtaaibos (Afrikaans)	/	/	/	PRE 1004267
*Bauhinia galpinii* N.E.Br. (Syn *Bauhinia galpinii* var*. ungulata* L.) (Fabaceae)	Pride of de kaap (English), vlam-van-die-vlakte (Afrikaans)	Gastrointestinal disorder, infertility, amenorrhoea, inflammation, and infectious diseases [16]	Leaves, bark, and seed	Antibacterial, and antioxidant, antimutagenic, cytotoxic activities [17, 18]	PRU 28944
*Bauhinia bowkeri* (Harv.) A. Schmitz (Fabaceae)	White Bauhinia (English), Kiebeesklou (Afrikaans), umdlandlovu	Gastrointestinal infection, induce vomiting, bathing, and steaming [19]	Leaves and bark	Anti-inflammatory, antibacterial, and antifungal activity [19]	PRU 127998
*Bauhinia variegata* L. (Fabaceae)	Mountain ebony, butterfly tree, orchid tree (English)	Diarrhoea, dysentery, goitre, diabetes [20]	Leaves and bark	Anti-inflammatory [21], Immunomodulatory [22]	PRU 38533
*Brachylaena transvaalensis* E. Phillips and Schweick (Asteraceae)	Forest silver-oak (English), vaalboom (Afrikaans), mufhata (Venda), iPhahla (Siswati)	Diarrhoea [23]	Leaves and bark	Antimicrobial activity [24]	PRU 126858

PRU: H.G.W.J. Schweickerdt Herbarium, University of Pretoria; PRE: Pretoria National Herbarium, South African National Biodiversity Institute, Pretoria; and /: not reported.

**Table 2:** Minimum inhibitory concentration (in mg/mL), cytotoxicity, and selectivity index (in brackets) of acetone plant extracts.

Plant	*E. coli*25922	*E. coli*35218	*E. coli* O157: H7	*S.* enteritidis	*B. cereus*	*P. aeruginosa*	*S. aureus*	*E. faecalis*	*S.*Typhimurium	LC_50_ (mg/mL)
*Searsia pendulina*	0.08 (0.37)	0.05 (0.56)	0.06 (0.48)	0.06 (0.44)	0.33 (0.09)	0.09 (0.31)	0.06 (0.43)	0.06 (0.45)	0.08 (0.37)	0.03 ± 0.00^*∗∗∗∗*^
*Searsia leptodictya*	0.08 (1.33)	0.06 (1.62)	0.08 (1.33)	0.08 (1.33)	0.16 (0.67)	0.09 (1.09)	0.07 (1.41)	0.13 (0.80)	0.16 (0.67)	0.11 ± 0.00^*∗∗∗∗*^
*Searsia lancea*	0.04 (5.82)	0.08 (2.55)	0.09 (2.19)	0.13 (1.53)	0.03 (6.11)	0.04 (4.36)	0.04 (5.09)	0.05 (3.52)	0.06 (3.05)	0.20 ± 0.02^*∗∗∗∗*^
*Searsia batophylla*	0.11 (1.32)	0.08 (1.90)	0.18 (0.82)	0.08 (1.90)	0.21 (0.63)	0.08 (1.80)	0.08 (1.71)	0.20 (0.73)	0.08 (1.90)	0.15 ± 0.01^*∗∗∗∗*^
*Bauhinia galpinii*	0.08 (**14.48**)	0.08 (**14.48**)	0.08 (**14.48**)	0.04 (**25.18**)	0.32 (3.37)	0.20 (4.83)	0.10 (**10.86**)	0.14 (7.90)	0.13 (8.69)	>1
*Bauhinia bowkeri*	0.07 (8.46)	0.08 (6.34)	0.09 (5.19)	0.01 (**50.75**)	0.27 (1.83)	0.10 (4.96)	0.06 (7.61)	0.15 (3.36)	0.06 (7.61)	0.51 ± 0.04^*∗∗∗∗*^
Gentamicin	0.005	0.005	0.001	0.010	0.005	0.002	0.005	0.005	0.002	nd
Doxorubicin	nd									0.01 ± 0.00

nd = not determined, bold values indicate significant SI values, and ^*∗∗∗∗*^*p* < 0.0001.

**Table 3:** Minimum inhibitory concentration (in mg/mL) of different extracts against *Escherichia coli* ATCC 25922.

Plant name	Extracts				
	Methanol/water	Methanol/DCM	Hot water	Ethanol	Acetone
*Searsia pendulina*	2.50	0.63	0.63	1.25	0.08
*Searsia leptodictya*	0.16	1.25	0.63	1.25	0.08
*Searsia gueinzii*	1.14	2.08	2.50	1.87	0.37
*Searsia lancea*	0.24	0.29	0.63	0.08	0.04
*Searsia batophylla*	0.32	0.29	ND	0.47	0.11
*Brachylaena transvaalensis*	1.25	1.25	0.63	0.83	0.63
*Bauhinia galpinii*	1.25	1.25	0.63	0.83	0.08
*Bauhinia bowkeri*	1.25	2.50	1.14	0.63	0.07
*Bauhinia variegata*	0.32	0.63	0.32	0.47	0.21
Gentamicin	0.005				

**Table 4:** Percentage of biofilm inhibition and eradication by acetone plant extracts.

Extracts	Biofilm formation inhibition (%)						Biofilm eradication					
	*E. coli*35218	*S. typhimurium*	*B. cereus*	*P. aeruginosa*	*E. faecalis*	*E. coli* 25922	*E. coli*35218	*S. typhimurium*	*B. cereus*	*P. aeruginosa*	*E. faecalis*	*E. coli*25922
*S. pendulina*	22.53	123.96	77.54	78.06	31.65	81.63	−83.59	25.03	98.22	−99.60	−25.77	40.29
*S. leptodictya*	13.31	112.30	72.89	111.47	21.20	124.28	−74.90	−7.93	69.15	79.85	−47.34	−74.46
*S. lancea*	6.46	65.44	22.67	60.02	24.14	76.97	−74.94	1.55	51.20	−199.40	−75.07	−95.23
*S. batophylla*	9.35	110.59	65.70	110.65	94.81	85.99	−35.39	−68.31	55.62	−224.57	34.44	−270.56
*B. galpinii*	0.85	91.71	26.20	71.06	78.04	54.60	11.13	13.13	42.50	−213.29	−27.88	−253.80
*B. bowkeri*	6.26	78.27	73.52	108.47	92.94	124.35	−84.19	32.82	70.29	−24.33	−16.56	−39.49
Gentamicin	81.88	103.66	100.05	100.76	74.73	105.26	62.27	74.25	53.79	73.87	67.93	51.93

## Data Availability

The original data can be obtained from the corresponding author upon request.

## Supplementary Materials

Supplementary Materials
*S1*. Pictures of some of the plants investigated.
