# Supplementary material for: Anti-Biofilm, Antibacterial, and Anti-Quorum Sensing Activities of Selected South African Plants Traditionally Used to Treat Diarrhoea
Source: Evid Based Complement Alternat Med. 2022 Sep 28;2022:1307801. doi: 10.1155/2022/1307801 (PMC9534605; doi:10.1155/2022/1307801)
Supplement: Supplementary Materials — S1. Pictures of some of the plants investigated. [file 1307801.f1.docx]

**Supplementary material**

**S1:** Pictures of some of the plants investigated
